# The Diversity and Distribution of Fungi on Residential Surfaces

**DOI:** 10.1371/journal.pone.0078866

**Published:** 2013-11-01

**Authors:** Rachel I. Adams, Marzia Miletto, John W. Taylor, Thomas D. Bruns

**Affiliations:** Plant & Microbial Biology, University of California, Berkeley, California, United States of America; University of Minnesota, United States of America

## Abstract

The predominant hypothesis regarding the composition of microbial assemblages in indoor environments is that fungal assemblages are structured by outdoor air with a moderate contribution by surface growth, whereas indoor bacterial assemblages represent a mixture of bacteria entered from outdoor air, shed by building inhabitants, and grown on surfaces. To test the fungal aspect of this hypothesis, we sampled fungi from three surface types likely to support growth and therefore possible contributors of fungi to indoor air: drains in kitchens and bathrooms, sills beneath condensation-prone windows, and skin of human inhabitants. Sampling was done in replicated units of a university-housing complex without reported mold problems, and sequences were analyzed using both QIIME and the new UPARSE approach to OTU-binning, to the same result. Surfaces demonstrated a mycological profile similar to that of outdoor air from the same locality, and assemblages clustered by surface type. “Weedy” genera typical of indoor air, such as *Cladosporium* and *Cryptococcus,* were abundant on sills, as were a diverse set of fungi of likely outdoor origin. Drains supported more depauperate assemblages than the other surfaces and contained thermotolerant genera such as *Exophiala*, *Candida*, and *Fusarium*. Most surprising was the composition detected on residents’ foreheads. In addition to harboring *Malassezia*, a known human commensal, skin also possessed a surprising richness of non-resident fungi, including plant pathogens such as ergot (*Claviceps purperea*). Overall, fungal richness across indoor surfaces was high, but based on known autecologies, most of these fungi were unlikely to be growing on surfaces. We conclude that while some endogenous fungal growth on typical household surfaces does occur, particularly on drains and skin, all residential surfaces appear – to varying degrees – to be passive collectors of airborne fungi of putative outdoor origin, a view of the origins of the indoor microbiome quite different from bacteria.

## Introduction

Although only as much as 0.7% of the global land-surface is covered in buildings [1], Americans spend on average about 87% of their time indoors, and most of it, 69%, in their homes [2]. Typically, fungi are of interest only when buildings are damaged by water entering the building due to leaks in the envelope, plumbing failures, or condensation. Under these conditions, indoor fungal growth can cause or exacerbate disease including asthma, primarily via fungal toxins and allergens [3]. Traditionally, fungi are identified by observing their morphology directly from captured spores or after cultivation. These morphological-based methods have produced a list approximately 90 species thought to be common and important players indoors – molds and yeasts such as those in the genera *Alternaria*, *Aspergillus*, *Cladosporium*, *Penicillium*, *Rhodotorula*, and *Wallemia* [4], [5]. These indoor fungi tend to be saprobic taxa with particular niches within the home that are dependent on substrate and water availability. For example, *Rhodotorula mucilaginosa* is common in moist rooms such as bathrooms and the more xerophilic *Aspergillus fumigatus* is associated with drier areas such as living rooms, while *Cladosporium* is ubiquitous throughout residences [6], [7].

Recently, nucleic acid sequencing has been applied to dust in indoor air from damaged and undamaged buildings to survey both fungi and even smaller microbes indoors, bacteria. These studies have not only greatly expanded the number of microbial taxa known from indoor air, they can be synthesized to a general hypothesis about the structure of indoor air microbial communities: indoor fungi represent a subsample of the outdoor fungal community with modest or little indication of internal growth [8]–[11], whereas indoor bacteria are more strongly influenced by the inhabitants of the buildings [12]–[17]. Fungal studies have primarily focused on spores, yeast cells, and fragments of these propagules and hyphae that are airborne or settled from air. For example, our previous work showed that airborne fungal assemblages collected from passively settled dust did not differ between rooms such as kitchens, bathrooms, bedrooms, and living rooms, even though they have obvious differences in uses, water sources, and potential substrates for fungal growth [10]. On the other hand, bacterial studies in homes have more often included samples of surfaces and the skin of building inhabitants as indicators of indoor bacterial communities [13], [17].

To identify possible areas of the indoor environment where fungi grow and could contribute to airborne assemblages through aerosolization in non-water damaged buildings, we compared fungi on three surface types to those fungi that we had previously detected in passively settled airborne dust [10]. Knowing that fungal growth is largely water-limited in buildings, we searched for sites that are both periodically wet and commonly found across different residences in order to test the hypothesis that the indoor fungal assemblages on surfaces are not an important source for indoor airbone fungal assemblages, as they are for bacteria. We selected three types of surfaces: windowsills, drains, and the forehead of inhabitants.

Previous work has suggested different fungal communities across these surface types. On the windowsills, which are wet by condensation, we expected to see the common indoor molds mentioned above [4]. In the drains, which by definition are wet, we hypothesized that we would see simple communities due to the reported domination of *Fusarium* compared to taxa such as *Penicillium* and *Mucor* [18], and we expected to see different communities structured by the particular nutrient environment of the drain type (kitchen, bathroom sink, bathtub). Finally, we predicted the forehead skin mycobiota to be dominated by *Malassezia*, a typical resident of human skin that can be a cause of dandruff, but also can harbor other, less frequently encountered taxa such as *Candida* and *Aspergillus* [19], [20]. While we did detect the presence of these known fungal associates of surfaces, overall we found the influence of outdoor air to be dominant.

## Materials and Methods

### Study site and sample collection

**Figure 1:**
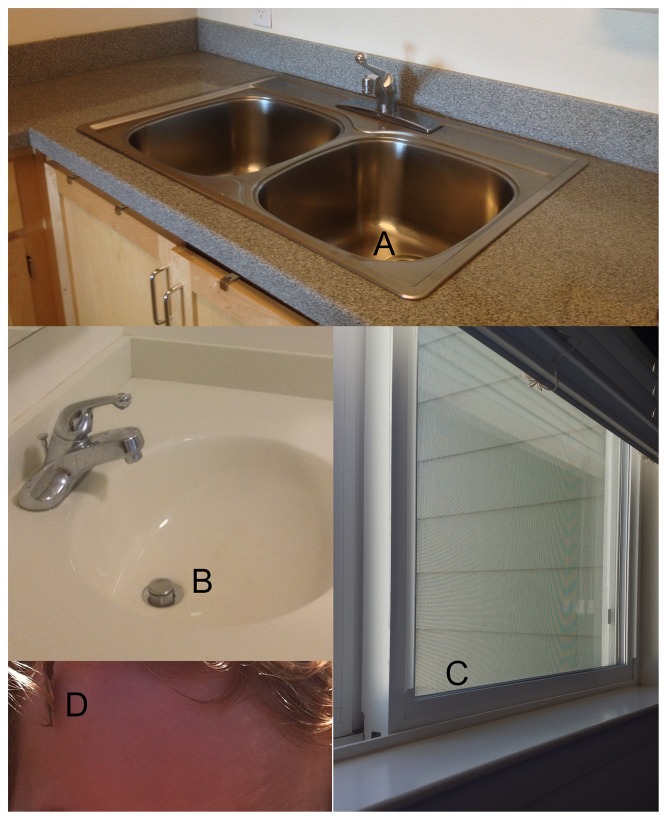
The Study Sites for Surveying Fungi in Residential Units of a University Housing Complex. Pictured are three surface types on what cotton-tipped swabs collected material for fungal analysis. Biofilms were collected on three types of drains: kitchen sinks (A), bathroom sinks (B), and bathtub drains. From the upward-facing edge of windowsills in kitchens, living rooms, bedrooms (C), and bathrooms, dust was also collected. Skin samples were taken from the forehead (D). Fungi on these surfaces were compared with each other and with passively settled indoor and outdoor dust from the same sampling locality [10].

We sampled areas of potential fungal growth in areas of a residence that are open to the living space, not regions hidden behind walls: drains in the kitchen sink, bathroom sink, and bathtub; the lower upward-facing edge of windowsills in the kitchen, living room, bedroom, and bathroom; and the foreheads of all residents of the unit (Figure 1). Sampling was replicated in 11 units of a university housing complex, in which all residences have identical construction materials and similar floor plans and which does not allow cats or dogs, as previously described [10]. To sample surfaces, sterile, nucleotide-free water was used to moisten a cotton swab, and the surface was rubbed for 5−10 seconds. We did not observe any visible fungal growth in these residences outside of drains. Swabs from home surfaces were stored in individual glass vials except for the forehead swabs, which were pooled for all residents of a unit to protect volunteer confidentiality. Swabs were stored frozen at −80 °C until nucleotide extraction. Additionally, residents completed a survey on the characteristics and typical use of their unit to provide information on: the number of bedrooms, bathrooms, and residents, the age of the unit, the presence of houseplant(s) and the use of a humidifier. Other parameters (frequency of cooking, cleaning, occupancy, and window opening) were invariable and therefore excluded from the analysis. Swab surveys were conducted twice, once in August 2011 in 11 units and again in January 2012 for 8 of those same units. The sampling protocol was regulated by the University of California’s Committee for the Protection of Human Subjects, Protocol ID #2011-03-2947, and approved by both the Village Residents Association for the housing complex on May 18, 2011 and the Residential and Student Service Programs of the University on July 25, 2011.

### DNA processing and bioinformatics analysis

Genomic DNA extraction relied on the MoBio PowerSoil Kit (Carlsbad, CA, USA) with some modifications following Fierer et al. [21]. Using scissors that had been immersed in ethanol and flamed, the cotton head of the swab was cut directly into the provided PowerBead tubes containing solution C1. The tubes were incubated at 65 °C for 10 minutes and vortexed horizontally for 10 minutes, after which manipulations followed the recommended protocol. The internal transcribed spacer (ITS) region 1 of the nuclear ribosomal coding cistron was targeted with primers ITS1F and ITS2 [22], [23]. The forward primer contained the 454 Fusion Primer A-adaptor and an 8-basepair MID barcode unique for each environmental sample, while the reverse primer contained the B-adaptor for the pyrosequencing. PCR amplifications of both samples and negative controls were was performed in triplicate, pooled, purified using the AMPure magnetic beads (Beckman Coulter Genomics, Danvers, MA), quantified using the Qubit fluorometer (Invitrogen, Carlsbad, CA, USA), and combined at a 50 ng equimolar concentration for downstream sequencing; full details on amplicon library preparation have been described previously [10]. Environmental samples were split across three different 1/8^th^ 454 FLX+ picotiter plates, which were sequenced at two different sequencing facilities, one plate at the Duke Institute for Genomic Sciences and Policy (Durham, NC) and two plates at the University of Illinois at Urbana-Champaign’s W.M. Keck Center for Comparative and Functional Genomics. Information on all the processed samples across the experimental units, including the MIDs, are detailed in Table S1.

Initially, sequences were bioinformatically processed with QIIME [24], and in the process of writing the paper, UPARSE [25] was released. UPARSE is a new method aimed at clustering globally-trimmed sequences into operational taxonomic units (OTUs) with a focus on reducing OTU inflation. Although the number of OTUs dropped from 1,305 taxa in QIIME (Protocol S1) to 966 in UPARSE, the qualitative outcomes are identical, corroborating the common observation that beta-diversity conclusions tend to be less sensitive than alpha diversity to “noisy” reads such as those generated by sequencing error or chimera formation [26]. Results are reported based on analysis with UPARSE. We followed the recommended pipeline [27], with setting a truncation length of 150bp and a maximum expected error of 0.5 for individual sequences and removing singletons. Chimeras were checked against the UNITE fungal database [28] version released August 26, 2013, and this database was also used to assign taxonomy based on the BLAST algorithm. Raw sequences files and accompanying metadata have been deposited into the NCBI SRA under accession SRP029981, and representative sequences of the final OTUs have also been deposited into GenBank with accession numbers KF221245-KF222494.

In order to estimate fungal biomass, we employed a Real-Time PCR System (Applied Biosystems 7300, Carlsbad, CA, USA). PCR samples contained 5 µl genomic DNA extract, 10 µl iTAQ SYBR Green Supermix with ROX (Bio-Rad, Hercules, CA, USA), 0.2 µl of 100 mg ml^−1^ BSA, 0.15 µl of each 50 µM forward and reverse primer, and water to 20 µl. Universal fungal primers FF2 and FR1, which amplify a 425-bp region of the small subunit rRNA, were used [29]. The standard curve was generated using a 5-fold dilution series of *Penicillium purpurogenum* spores spanning 5−50,000 spores. The thermocycler profile began with denaturation at 95 °C for 3 minutes, followed by 40 cycles of 95°C for 15s and 60 °C for 60s (the later being the data collection stage), and ending with a dissociation curve of 95 °C for 15s, 60 °C for 30s, and 95 °C for 15s. We checked for inhibition in a subset of samples by including a known amount of *P. purpurogenum* spores with an environmental sample. If the quantified estimate was less than the known input of spores, it would indicate the genomic extract contain inhibitors to amplification. No indication of inhibition was observed for any of the surface types, so all samples were run with undiluted genomic DNA. The qPCR for each sample were done in triplicate, and the mean value used for analysis. Due to the inherit limitations of qPCR to estimate biomass, we treat results as indicators of relative differences of fungal material across samples.

### Community analyses

Fungal taxa present in negative controls (Table S2) were excluded from the study samples. The taxa in negative controls (n = 67) were not particular abundant or frequent (Table S2), and thus, excluding all taxa present in the negative controls is a conservative approach that avoids counting possible contaminant taxa with scant effect on sample assemblages. Two areas of swabbing, the bathtub tiles and the wall behind the kitchen stove (a possible site of condensation due to cooking), were excluded due to poor sample amplification and low number of reads for those that did amplify. Finally, we only included those samples that contained at least 100 sequences and rarefied the community table to that sequence number prior to all analyses. Although a low coverage number, it has been shown to be sufficient for identifying differences in microbial communities [30].

For comparisons across samples, the community table was analyzed in R [31] to implement statistical tests of richness, compositional differences, and isolation by distance. We visualized differences in community composition using nonmetric multidimensional scaling (NMDS) and examined factors including composition with ADONIS, a permutational multivariate analysis of variance [32]. Differences in community composition used both the Jaccard and Morisita-Horn indices, which rely on presence-absence and abundance data, respectively. As mentioned, samples were sequenced on three different plates across two sequencing facilities, so we report results of measured environmental elements after factoring out the effect of sequencing run [following 33] in a multifactorial model as implemented with ADONIS [32]. Statistical effects of geographic differences are based on mantel correlations.

## Results

### Fungal community composition: links to indoor air and indications of growth

**Figure 2:**
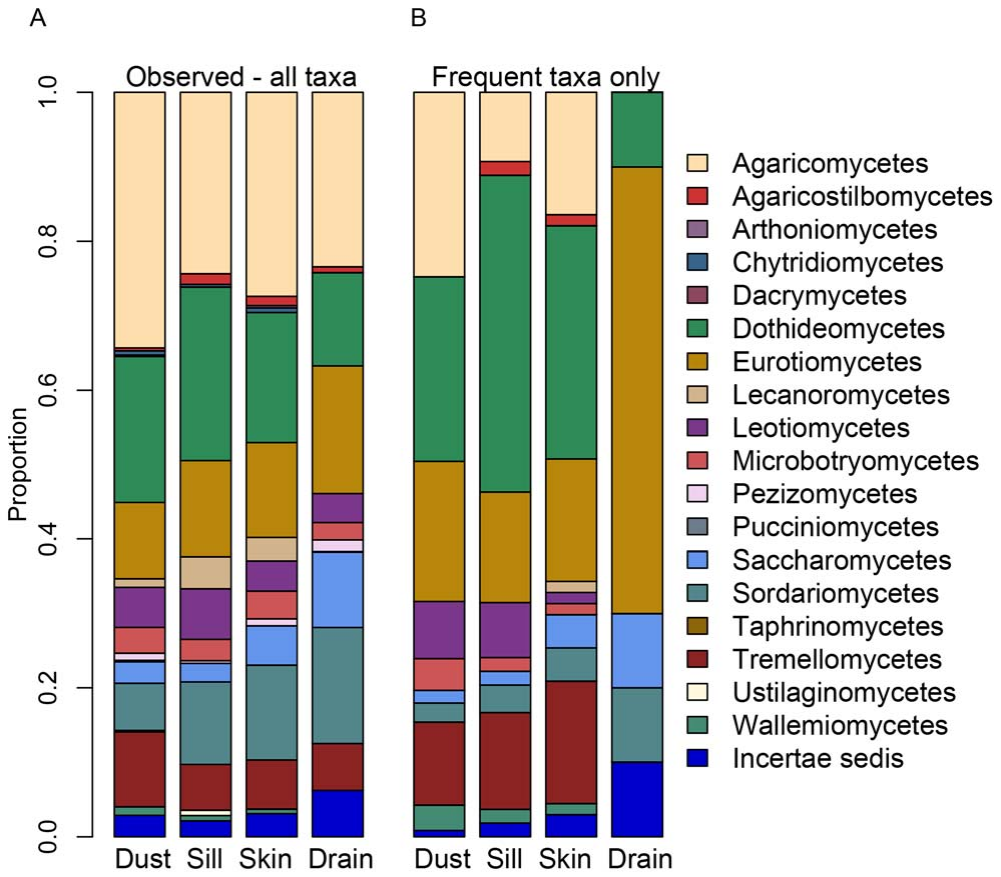
Classes of Fungi Represented across Different Parts of the Built Environment. Panel A: Eighteen different classes of fungi were represented on sills, drains, and skin. Both the broad composition of fungal taxa as well as their relative abundances are similar across the different surfaces, as well as to passively-settled dust from the outdoor air at the same location [10]. Particularly abundant are Agaricomycetes (mushrooms, broadly), Dothidiomyetes (including molds and plant pathogens), Eurotiomycetes (yeasts), Sordariomycetes (including molds), and Tremellomycetes (plant and soil-associated). Panel B: Drains stand distinct from other surfaces and dust samples when considering only those frequent taxa present in at least 10% of each sample type. Drains appear less rich and are relatively overrepresented by Eurotiomycetes (*Exophiala*), Microbotryomycetes (*Rhodotorula*), and Sordariomycetes (*Fusarium*).

Fungal community composition on indoor surfaces was extremely diverse with respect to taxonomic representation and ecological role (Table 1; Figure 2). Many of the most common taxa overlapped with those known from culture-based surveys of the indoor environment: *Cladosporium*, *Cryptococcus*, *Penicillium*, *Candida*, *Malassezia*, *Phoma*, *Exophiala*, *Rhodotorula*, *Wallemia*, and *Fusarium.* However, the diversity within generic level clades, for example, *Cladosporium*, *Cryptococcus*, *Exophiala,* was greater than expected. Moreover, there were abundant taxa, such as, *Botryotinia fuckeliana* and *Kondoa aeria,* which had not been frequently detected from culture-work on the built environment. Many taxa, although not the most common ones, only matched other unnamed sequences in the database and could therefore not be identified.

**Table 1:** Identify of common fungi and their frequency across samples, as percentages.

	% Match^1^	Total (101)	Sills (41)	Skin (32)	Drains (28)
*Cladosporium cladosporioides*	100	58.4	80.5	78.1	3.6
*Cryptococcus sp.*	100	58.4	75.6	81.3	7.1
*Cladosporium sp.*	98.7	54.5	78.0	65.6	7.1
*Cryptococcus sp.*	100	52.5	65.9	71.9	10.7
*Cryptococcus sp.*	100	51.5	70.7	65.6	7.1
*Cladosporium aphidis*	100	50.5	68.3	68.8	3.6
*Stemphylium sp.*	100	49.5	70.7	53.1	14.3
*Lewia sp.*	100	48.5	61.0	65.6	10.7
*Botryotinia fuckeliana*	100	46.5	58.5	65.6	7.1
*Cryptococcus sp.*	100	42.6	26.8	71.9	32.1
*Sclerococcum sp.*	95.9	35.6	41.5	56.3	3.6
*Verrucocladosporium dirinae*	94.8	33.7	53.7	31.3	7.1
*Coniosporium*	96.7	30.7	39.0	40.6	7.1
*Kondoa aeria*	99.3	29.7	39.0	40.6	3.6
*Cladosporium cladosporioides*	100	28.7	39.0	21.9	21.4
*Cryptococcus macerans*	100	28.7	22.0	56.3	7.1
*Malassezia sp.*	100	27.7	0.0	78.1	10.7
*Penicillium spinulosum*	100	27.7	17.1	62.5	3.6
*Penicillium sp.*	100	25.7	12.2	59.4	7.1
*Debaryomyces sp.*	100	24.8	12.2	56.3	7.1
*Penicillium sp.*	100	24.8	12.2	43.8	21.4
*Rhodotorula sp.*	98.0	22.8	14.6	50.0	3.6
*Candida parapsilosis*	100	20.8	2.4	40.6	25.0
*Trichosporon dermatis*	100	20.8	7.3	28.1	32.1
*Cryptococcus macerans*	98.6	19.8	9.8	46.9	3.6
*Exophiala lecanii corni*	100	17.8	4.9	15.6	39.3
*Phoma herbarum*	100	14.9	12.2	15.6	17.9
*Candida parapsilosis*	97.4	14.9	0.0	37.5	10.7
*Claviceps purpurea*	98.7	13.9	4.9	37.5	0.0
*Exophiala dermatitidis*	100	12.9	0.0	6.3	39.3
*Exophiala sp.*	99.3	8.9	0.0	3.1	28.6
*Exophiala lecanii corni*	97.6	6.9	0.0	0.0	25.0
*Exophiala sp.*	100	6.9	0.0	0.0	25.0
*Fusarium solani*	98.0	6.9	2.4	0.0	21.4
*Ochroconis constricta*	100	5.9	0.0	0.0	21.4

Notes: ^1^ Match to sequences in the UNITE fungal database [28], version released August 26, 2013.

Taxonomic diversity on all surfaces matched the class-level composition previously reported from passively collected outdoor dust at the same locality [10] (Figure 2). Across all the surface types and the airborne samples, Basidiomycota such as Agaricomycetes, Microbotryomycetes, and Tremellomycetes, and Agaricomycota such as Dothideomycetes, Eurotiomycetes, Leotiomycetes, Saccharomycetes, and Sordariomycetes, were well represented. As a broad snapshot, the composition of fungi on the different surfaces by taxonomic class showed strikingly similarity to indoor and outdoor air samples from the same sites reported previously, and to each other regardless of surface type.

Obvious differences in composition across surfaces are only apparent when comparison were limited to frequent taxa, those that are found in at least 10% of each sample type and then only drains look distinct from other surfaces (Figure 2). When looking at only these frequent taxa, the drain samples had a greater percentage of Eurotiomycetes (*Exophiala*), Saccharomycetes (*Candida*), and Sordariomycetes (*Fusarium*). In particular, it is black yeasts like *Exophiala* and *Ochroconis,* that were commonly detected in drains (Table 1). On the other hand, Agaricomycetes, Dothideomycetes, Glomeromycota, Lecanoromycetes, and Taphrinomycetes and many taxa common on sills were also common in forehead samples (Table 1). Skin samples were dominated by a suite of taxa that are only known from non-human and non-animal habitats, fungi including *Cladosporium*, *Penicillium spinulosa*, *Rhodotorula*, and *Claviceps purpurea*. Of these, the one that particularly stands out is *Claviceps purpurea*, the ergot fungus known for its pathogenic relationship with grains. Ergot was common on both windowsills and foreheads in the summer. The only known human-associated fungi that were frequently found were *Candida parapsilosis* and *Malassezia* sp. (Table 1).

### Biogeographic patterns among indoor surfaces

Bioinformatic processing resulted in a total of 59,201 individual fungal sequence reads clustered into 966 OTUs. Observed accumulation curves showed that increased sampling both within and across residential units would likely detect further fungal OTUs, particularly for windowsills (Figure S1). Although overall richness was high, few fungal taxa were commonly found in multiple samples, and the median observed richness in a sample was 32 OTUs. The vast majority of taxa were rare in two ways: abundance (number of reads) and frequency (how often they appear in a sample, Figure S2). For instance, the median abundance for all taxa is approximately two individual reads, and 147 taxa (15%) are represented by one or two reads. Likewise, 35% of the taxa appear in only one sample, and only 11% of the OTUs appear in at least 10 of the 101 samples.

As sampled by surface swabs, drains appear less rich than sills and foreheads: the median observed rarified richness was 6 taxa in drains, 19 in sills, and 32 on skin. Shannon and Simpson diversity indices also indicate that drains are less diverse than the other surface types (Table S3). Within surface types, bathroom sinks (n = 14) show fewer taxa than kitchen sinks (n = 5) (Fig S3). Plus, fungal richness of windowsills decreased as the number of bedrooms or residents increased (Figure S3). There were no observed differences across drains, sills, and skin on fungal richness by other unit or resident characteristics (such as presence of humidifier or houseplant(s)).

Biomass estimates showed a clear pattern: skin showed a consistent signal for low fungal biomass, and sills and drains were far more variable but generally showed much higher fungal biomass. The mean spore equivalents on skin were 114 *P. purpurogenum* spores (σ^2^ = 187). Windowsills showed high average biomass (µ = 906,608 spore equivalents, σ^2^ = 2,196,719) and ranged from 14 to 9,834,754 spore equivalents. Within drains most samples were of moderate biomass (µ = 1,430 *P. purpurogenum* spore equivalents, σ^2^ = 3,383). However, three drains (one kitchen, two bathroom sinks) samples were extreme outliers, even after repeated amplifications, demonstrating biomass of >70,000,000 spore equivalents. The high-biomass drain samples appeared in three different units, while the high-biomass windowsills samples all came from a single unit. Beyond surface type, the only significant trend we observed in abundance was an increase in fungal biomass with increasing number of residents.

**Figure 3:**
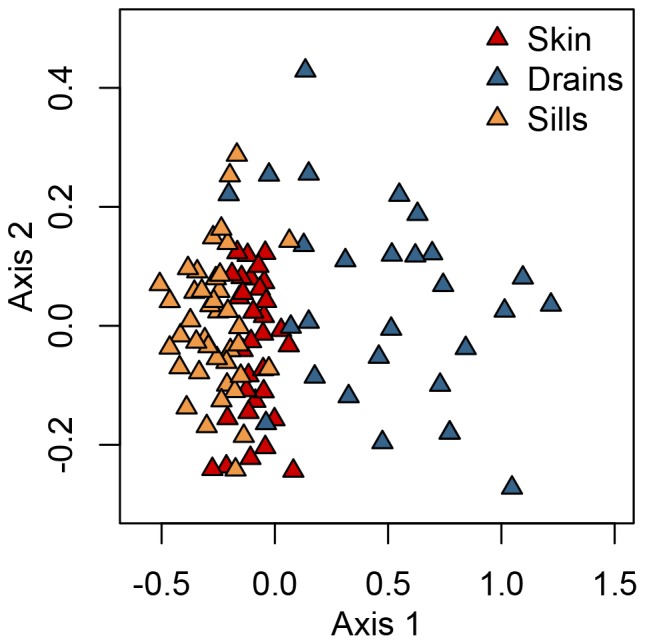
Visual representation of the fungal composition on different surface types in residences using nonmetric multidimensional scaling (NMDS) based on the Morisita-Horn (abundance-based) index. The composition of fungi cluster by surface type, with drains showing higher variation across samples than skin and sills (NMDS stress = 0.13).

Visualization of community composition showed clustering by surface type and this result was independent of whether read abundance of taxa (Figure 3; ADONIS p<0.05) or simply presence-absence of taxa (Figure S4) were considered. Fungal samples on sills, skin, and drain swabs were distinct in composition space, although sills have more taxa in common with skin swabs than drains. In testing for differences in group variances, drains and skin show modest significant differences from each other (multivariate homogeneity of group dispersions [34] as implemented in vegan [32]; Tukey multiple comparisons of means p = 0.05), with drains more variable than skin swabs. After factoring out plate effects across the three different pyrosequencing runs (between 1−9% of the total variation), the residential unit explained the largest percentage of variation in fungal community composition (Table 2). Due to the dominant effect of unit, we only report those factors that had a significant effect after variation due to residential unit was accounted for. Thus, only surface sample type and room function (and no unit characteristics or behavioral factors of the residents in the unit, such as the number of bedrooms or the use of a humidifier) remains as significant predictors of fungal community composition.

**Table 2:** Predictive factors of fungal community composition on surfaces within residential apartments.

	All				Drains			
n	101				28			
	df	F-value	R^2^	p	df	F-value	R^2^	p
Type^1^	2	5.8	0.10	0.001	2	2.3	0.12	0.001
Residential Unit	10	1.7	0.14	0.001	10	2.0	0.51	0.001
Room function	3	1.6	0.03	0.01	--	--	--	ns
Residuals	83		0.67		14		0.35	
	Sills				Skin			
n	41				32			
	df	F-value	R^2^	p	df	F-value	R^2^	p
Residential Unit	10	1.9	0.36	0.001	8	1.5	0.34	0.001
Room function	3	1.6	0.09	0.03	--	--	--	--^2^
Residuals	26		0.48		21		0.55	

Notes: Community composition based on the Morisita-Horn index, with values are reported after factoring out sequencing plate effects. ^1^For all samples, type refers to drain, windowsill, or skin swab. For drains, type refers to bathroom sink, bathtub drain, or kitchen sink. ^2^Room function not included in model for skin only samples. ns  =  not significant. df  =  degrees of freedom.

Within drains, the largest predictor of fungal community composition was unit, followed by type (bathroom sink, bathtub drain, kitchen sink) (Table 2). For windowsills, unit was the dominant predictor community composition, followed by room function (kitchen, bedroom, living room, or bathroom). For skin, unit was the only significant predictor of community composition. Results were consistent when community composition was analyzed using the Jaccard index, a presence-absence based metric that would be more sensitive to rare taxa, but the factors explained slightly less of the variation in community composition (Table S4) than when using the Morisita-Horn index reported in Table 2.

Fungal composition in drains showed more continuity over time than windowsills. We compared the beta-diversity community distance of the same sampling location across time with community distance across space: for example, a drain sampled in the summer with its winter sample against all drains sampled in the summer. Variation within an individual drain across time was varied but significantly lower than variation across space (Figure S5). A windowsill, on the other hand, was no more likely to show a similar fungal composition across time as it was to another windowsills across space. For skin, no time comparisons were not possible, because skin swabs were only identified by unit, not to the individual.

We found some evidence of by distance in our samples, although we were constrained by low sample sizes when looking within sample types and seasons (Table S5). In particular, differences in fungal communities increased with geographic distance in the winter on both windowsills and foreheads.

## Discussion

### Indoor fungal residents

The question for this study was to determine if surfaces in buildings or on their occupants contributed to the fungal components of indoor air on a local scale, as has been shown for bacteria. The motivation came from our prior work in this housing complex, which showed that dispersal from the outdoor air dominated the signal of indoor air, that many of the common indoor fungi had a likely outdoor origin, and that dispersal limitation in fungi was the single most important factor structuring indoor air [10]. In the current study we show that, although the fungal assemblages on various surfaces in these homes are distinct, they are still largely dominated by fungi common in outdoor air. Fungal taxa that are likely growing on these surfaces (e.g., *Malassezia* on skin, *Exophiala* in drains – discussed later) and which are most expected to contribute to microbes to indoor air are but minor components of the indoor air (as these genera were the 65th and 29^th^, respectively, most frequent of 271 genera found in the indoor air from our previous study [10]).

Windowsills offer compelling examples of depositional areas. Many of the dominant fungal groups on windowsills (e.g. Agaricomycetes, Glomeromycetes, Lecanoromycetes, and Taphrinomycetes) are unlikely to be growing on the surfaces due to their generally defined plant- or soil-associated ecologies. On the other hand, members of the genus *Cladosporium* (Dothideomycetes) are often observed growing on windowsills and other sporadically moistened surfaces in houses. But *Cladosporium* were also amongst the most common fungi found in outdoor air at these same sites [10], and so their abundance here on sills could be explained primarily by deposition rather that growth. Deposition clearly explains their presence on skin samples, as there is no prior evidence of *Cladosporium* growth on healthy humans, and their frequency on sills is only marginally higher than that observed on skin. Taken together, results from these studies suggest that in healthy buildings without reported mold problems, indoor surfaces are not a dominant source for indoor fungi. In fact the reverse is true: surfaces appear to be primarily depositional environments that collect many of the same fungi common in outdoor and indoor air.

Nevertheless, we did detect spatial structure in our samples that was dependent on both the physical location (unit) as well as the surface type (sill, skin, and drain – Figure 3). This biogeographic structure of surfaces – absent from airborne samples of passively settled dust [10] – shows that different surface types are likely to be harboring different deposition patterns or growth. Drains show clear evidence of both. Compositional difference driven by deposition is shown by the skin-associated *Malassezia* that was detected in bathroom drains (sink and bathtub) but not kitchens. An explanation for the greater richness in kitchen drains compared to bathroom sinks (Figure S3) could involve a richer input of nutrients for fungi in kitchens than in bathrooms. On the other hand, endogenous grown most likely accounts for some of the unique patterns found in drains. The increased frequency and relative abundance of *Fusarium* and *Exophiala*, taxa that are thermotolerant, acidotolerant, oligotrophic, and able to utilize surfactants [35]–[38], suggests a distinct drain niche relative to the other surfaces. Our results indicate that the part of the indoor environment that is most compatible for microbial growth in healthy buildings, unsurprisingly, belongs to the plumbing system.

Skin is known to be a habitat for some fungi, but our results indicate that these fungi may not be a particularly abundant set of taxa relative to those that passively collect on skin. For example, recent work on the fungal associates of the human body has demonstrated the prominence of *Malassezia* and to a lesser extent *Aspergillus* and *Candida* [19], [20]. While we did identify *Malassezia* and *Candida parapsilosis* on foreheads (Table 1), they were not the dominant taxa, in either frequency or abundance, in our study. Some of the variation in relative contributions of taxa across studies could be based on primer selection, as taxonomic bias in PCR amplification is a known limitation of the nucleic-acid based approach [e.g. 39]. Regardless of primer choice, many studies find an unexpectedly high richness of fungi in and on the human body [40], particularly the feet [20], and this diversity is composed of fungi whose known ecologies make growth on humans unlikely. Our data are in agreement with these studies and support the hypothesis that exposed skin surfaces are passive collectors of environmental fungi, such that we humans appear to be walking through the thin microbial soup of air all the while randomly collecting fungi on our bodies [41] and clothing. Nothing makes this point as clearly as the ubiquity on foreheads of the plant pathogen *Claviceps purperea*, better known as ergot, which is known to occur on grasses growing in the salt marsh near the study site [42]. Although we detected many *Cryptococcus* yeasts that could theoretically be growing on skin, the genus is diverse, and most species are mycoparasitic and plant- or soil-associated. *Cryptococcus victoriae* was the most frequent taxon in outdoor air at these same sites [10], and *Crytococcus* are six out of the top 30 most common fungi in this study. Thus, there is little evidence that the abundant fungi on foreheads (Table 1), with the exception of *Malassezia* spp. and *Candida*, have originated from growth on humans.

This is not to say that shedding of human-associated microbes does not occur or that it is not medically relevant. *Malassezia* and *Candida parapsilosis* are picked up at least rarely in airborne dust and windowsills and more commonly in drains (Table 1), suggesting that shedding of fungi when skin is washed may occur more readily than skin flaking directly into the air. From a health perspective, it is aerosolization of fungal material, rather than direct contact, that typically confers the greater exposure risk [43], and exposure to fungal aerosols in moldy buildings has been linked to new-onset asthma in adults [44]. Aerosolization of fungi from water systems can also have implications for human health [45]. For example, *Fusarium* growing in sink drains is the likely source of contamination in contact lens solution that led to an outbreak of *Fusarium* keratitis [18], [46]. However, our work indicates that when looking at the entire fungal profile in healthy buildings, aerosolization of particular fungi from growth on surfaces is minor when compared to fungal input from outdoors.

### Fungal abundance and human behavior

We have recently shown that highly varied abundances across samples can result in a perceived reduction in richness for the highly-abundant samples [47]. The reduction in richness is related to the finite number of ITS molecules that can be sequenced from each equimolar PCR-amplified environmental sample. Where the biomass in the samples is low, the read depth for each sample is high, but where the biomass is high, the read depth is low. Thus, in a high biomass sample with abundant taxa, the rare taxa will be missed and the richness will appear lower than low biomass samples sequenced concurrently [47]. These potential biases are also in play in this study, where the higher fungal richness on foreheads compared to other surfaces (Figure S1) is likely caused by the lower fungal biomass of forehead samples. Thus, biomass is an essential piece of data for correctly interpreting results and inferring process from environmental surveys. Here biomass estimates indicated that fungal presence on foreheads is rather limited, and coupled with the composition of fungi we found, most of the fungi on skin likely settled from the surrounding air. Similarly, the decreasing fungal richness as the number of residents increases (Figure S4) is likely caused by increasing biomass in these higher-occupied units. The reverse scenario is likely true for drain samples: the richness is likely underestimated because many of the dominant taxa (*Exophiala, Fusarium*, & others) are likely residents growing in this moist habitat resulting in higher fungal biomass. The solution to this problem is to increase the number of reads obtained per sample in high-throughput sequencing technology in order to saturate the accumulation curves.

While human behavior has no discernable effect on fungal community composition (Table 2), it may affect richness, and this aspect of the research deserves further investigation. For example, we found increased richness of windowsills in those units that reported occasional use of a humidifier. Humidification would be expected to increase condensation and therefore available water on cooler surfaces such as windows, and in this way could lead to localized fungal growth, although we did not observe higher fungal biomass in humidified units. Research has been done on how humidity affects bioaerosol viability but not, to our knowledge, bioaerosol concentrations. There are of course other aspects of resident behavior that either went unmeasured or were invariant, and the most important of these may be the absence of pets. Pets, particularly dogs, are a known source of microbes in homes, both through their resident communities and through the tracking of outdoor microbes to the indoors [13], [48].

### Comparisons with bacteria

Fungi and bacteria show vastly different source patterns on surfaces in the built environment. The results here indicate that known human-associated fungi contribute approximately 6% of the total fungi to non-human surfaces. In contrast, bacteria show a much higher percentage of human-association taxa on a variety of indoor surfaces. Five types of bacteria associated with humans made up 30% of surface samples in airplanes [49], and 20% of bacteria on kitchen surfaces are associated with the palm specifically [12]. Public restroom surfaces, excluding the floor, were at least 75% human-associated bacteria when those bacteria that were associated with not just skin but also gut, urine, and mouth [17]. Recently, swabs of surfaces in 40 residences indicates that approximately 80% of bacteria on surfaces in homes are human-associated [13]. Despite the differences in the prominence of humans as a source of microbes on indoor surfaces, studies of bacteria also show distinct assemblages on different surfaces types [17], and the least diverse bacterial communities associated with metallic surfaces around the kitchen sink, including the drain [12], as we found for fungi in this study.

Microbial assemblages in airborne samples show a similar trend to indoor surfaces with a higher frequency of human-associated bacteria compared to human-associated fungi. Our previous work on passively-collected airborne dust indicates that human-associated fungi make up approximately 3% of the total fungal community [10], and this is similar to that estimated from a global survey of vacuum dust [9]. However, human-associated bacteria comprise about 20% of all bacteria in indoor air of classrooms [14]. Meadow et al. [50] report a similar value for airborne bacteria in classrooms, with a max of 38% and mean of 7.8% of all bacterial sequences. Within residences, the bacteria on upper doorframes – surfaces specifically targeted to represent passive collectors of microbial assemblages in air – are approximately 30% human-originated [13].

These differences in the importance of human-associated bacteria versus fungi in the indoor environment are likely due to large differences in relative source strength between these two groups of microbes, as fungal population sizes on humans are much lower than bacterial populations [40]. In contract, fungal assemblages in outdoor air are large [51], and can be larger than those of bacteria [52], but proportionally the makeup of outdoor sources to human-associated assemblages is much larger for fungi than for bacteria. Thus it is not surprising that fungi from the outdoor environment swamp out the signature of human-associated fungi in the indoor environment, while bacterial indoor assemblages retain a stronger human-associated signature. What is perhaps surprising is that there is little evidence of any significant indoor fungal source for airborne assemblages such that the indoors appears to be largely an immigrate assemblage with dispersal and deposition from the outdoors overwhelming indoor growth. The issue of growth versus deposition has largely been unaddressed in bacterial studies, but this may ultimately prove to be a source of similarity across microbes if, as we suspect, most of the bacterial assemblages on surfaces turn out to represent passive deposition rather than *in situ* growth as they appear to be for fungi.

## Supporting Information

Figure S1 **Observed fungal richness accumulation curves for the three residential surface types.** Samples are pooled by type, and shaded areas represent the standard deviation around the mean. Drains (n = 28) appear less rich than sills (n = 41) and skin (n = 32) when compared to equal sequencing depths as represented by the number of amplicon sequences.
(TIF)

Figure S2 **Fungal taxa read abundance and frequency across samples.** Panel A: Taxa abundance distribution, showing that few taxa are represented by a large number of sequence reads while most are represented by a small number of sequences reads. Panel B: Similarly, most taxa appear in only a handful of samples and a more limited number of taxa are common across samples. The maximum number of samples is 101.
(TIF)

Figure S3 **Observed differences in fungal OTU richness within surface types across different seasons, room locations, unit characteristics, and resident behavior.** Shown are the significant factors affecting fungal richness on drains and windowsills. There were no differences observed for skins. The factors included: unit, room type, season, number of bedrooms, number of bathrooms, number of residents, age of unit, whether a humidifier was occasionally used, and whether a houseplant(s) were present. Within drains, bathroom sinks were less rich than kitchen sinks (anova – Table S3). Fungal richness of windowsills decreases with increasing number of bedrooms (anova, df = 2, Fvalue = 2.88, p = 0.06) and the number of residents (anova, df = 4, Fvalue = 2.77,p = 0.04). Letters indicate significant differences according to post-hoc Tukey Honest Significant Differences (p<0.05). Nonsignificant trends were based on tests of anova, p>0.05.
(TIF)

Figure S4 **Visual representation of the fungal composition on different surface types using nonmetric multidimensional scaling (NMDS) based on the presence-absence of taxa (Jaccard index).** As with the abundance-based data Morisita-Horn index, the three surface types cluster within types, with greater dispersion within drains.
(TIF)

Figure S5 **Variation of fungal community composition on drains and windowsills across time and space.** For drains, “time” is the mean dissimilarity in fungal community composition within a drain across seasons, while “space” is the dissimilarity across all drains within a season. Likewise for windowsills. Fungal communities within drains show more continuity over time than fungal communities on windowsills (2-sample t-test for drains: t = −2.62, df = 8.15, p = 0.03; for windowsills: t = −0.5, df = 14.25, p = 0.62).
(TIF)

Table S1 Summary of all the surface samples, including negatives, used in this study, with MID barcodes and sample location.
(XLSX)

Table S2 Fungal OTUs identified to genera in negative controls and removed from the study community table prior to analysis. Taxa marked with * were present in greater than 25% of all negative controls (n = 5), and taxa marked with ? were present as 1% or greater of total negative control sequences (n = 13).
(DOCX)

Table S3 Simpson and Shannon diversity indices for fungal richness on the different surface types. As with observed richness, drains are less diverse than sills and skin, but all three are significantly different from each other (anova: Simpson: df = 2, F-value = 24.53,p<0.01; Shannon: df = 2,F-value = 33.29,p<0.01; post-hoc TukeyHSD pairwise comparisons p<0.01). Bathroom sinks are less diverse than kitchen sinks (anova: Simpson: df = 2, F-value = 5.05,p = 0.01; Shannon: df = 2,F-value = 6.16,p<0.01; post-hoc TukeyHSD pairwise comparison between bathroom sinks and kitchen sinks p<0.01).
(DOCX)

Table S4 Predictive factors of fungal community composition on surfaces within residential apartments based on the (presence-absence) Jaccard Index, after the plate effect of the three sequencing runs were factored out.
(DOCX)

Table S5 Mantel correlations between geographic distance and fungal community distance.
(DOCX)

Protocol S1 Processing reads with QIIME and comparisons with UPARSE.
(DOCX)
