# Supplementary figures and images for: The Diversity and Distribution of Fungi on Residential Surfaces
Source: PLoS One. 2013 Nov 1;8(11):e78866. doi: 10.1371/journal.pone.0078866 (PMC3815347; doi:10.1371/journal.pone.0078866)

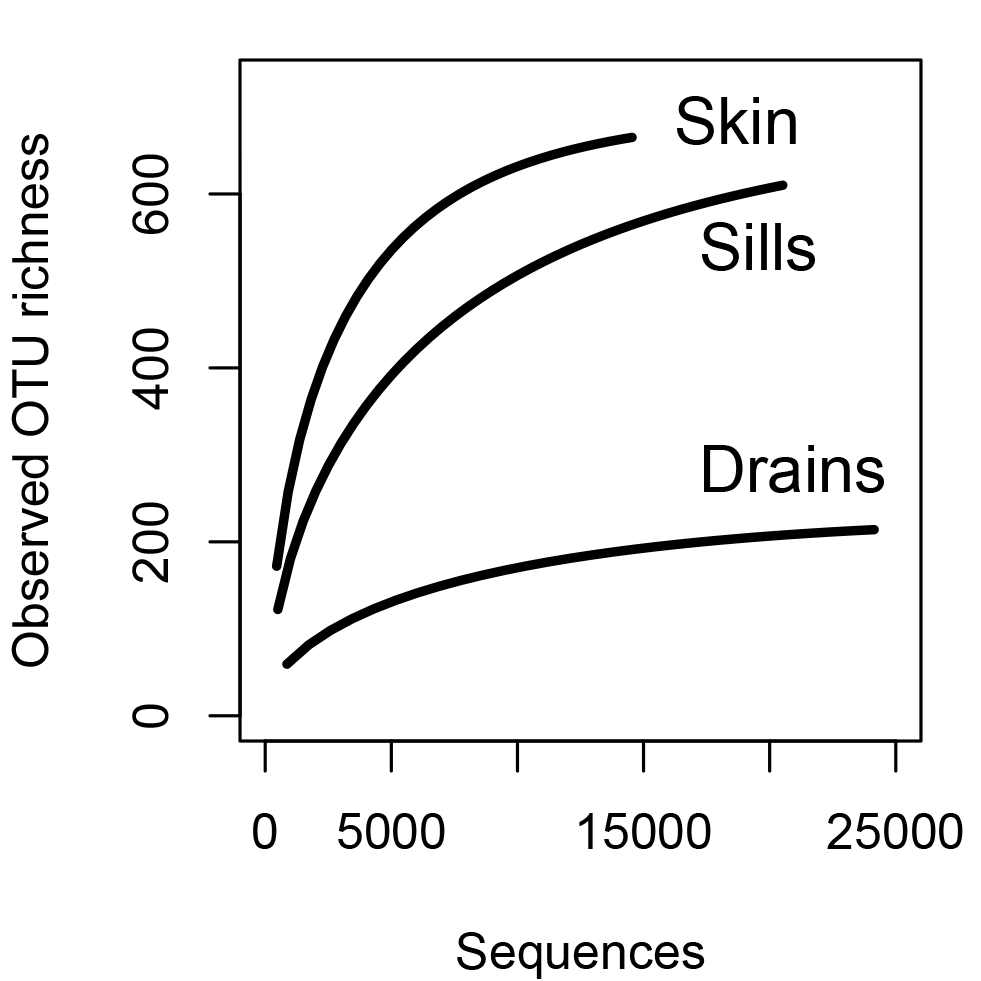

Supplement: Figure S1 — Observed fungal richness accumulation curves for the three residential surface types. Samples are pooled by type, and shaded areas represent the standard deviation around the mean. Drains (n = 28) appear less rich than sills (n = 41) and skin (n = 32) when compared to equal sequencing depths as represented by the number of amplicon sequences. (TIF) [file pone.0078866.s001.tif]

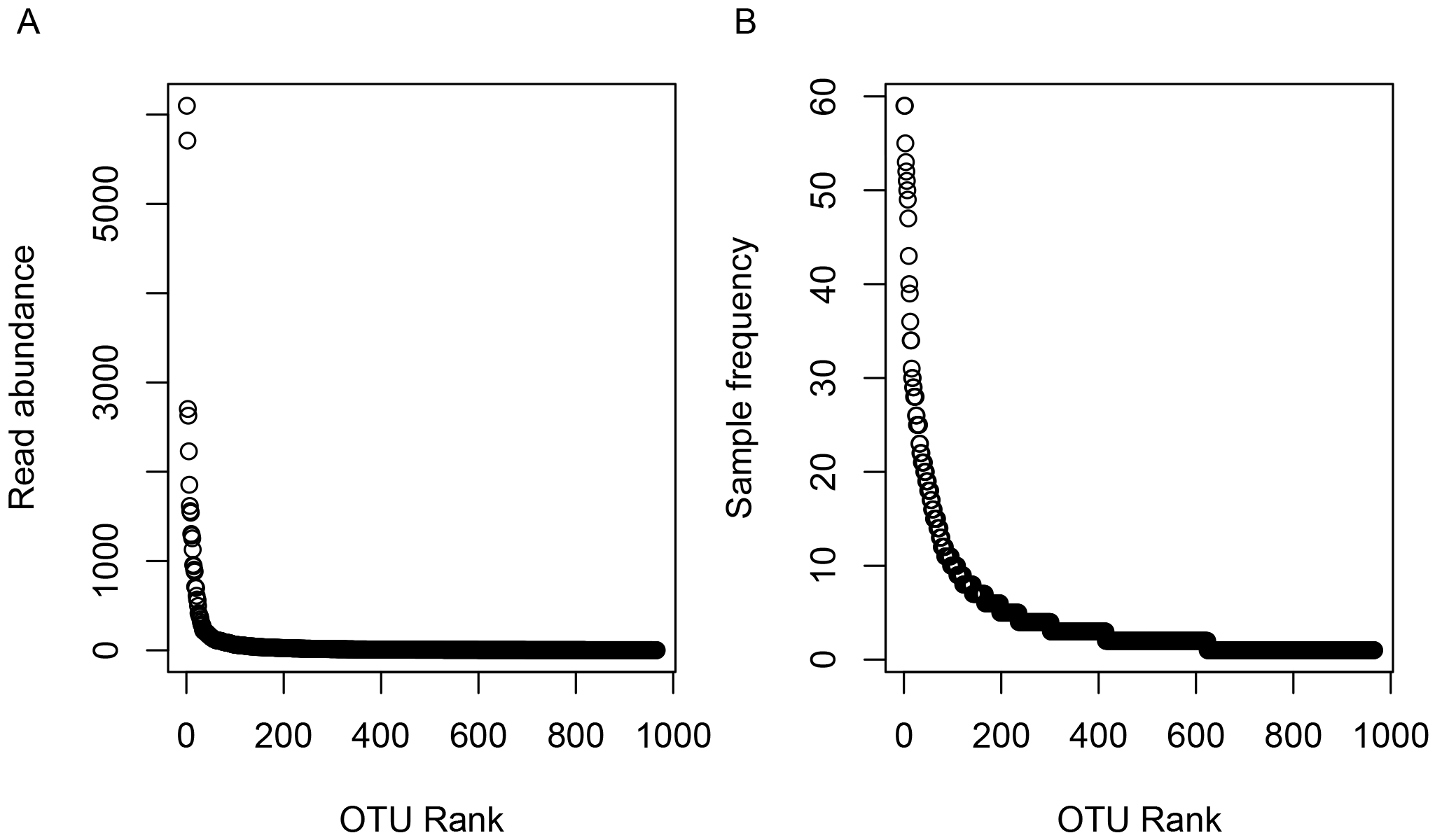

Supplement: Figure S2 — Fungal taxa read abundance and frequency across samples. Panel A: Taxa abundance distribution, showing that few taxa are represented by a large number of sequence reads while most are represented by a small number of sequences reads. Panel B: Similarly, most taxa appear in only a handful of samples and a more limited number of taxa are common across samples. The maximum number of samples is 101. (TIF) [file pone.0078866.s002.tif]

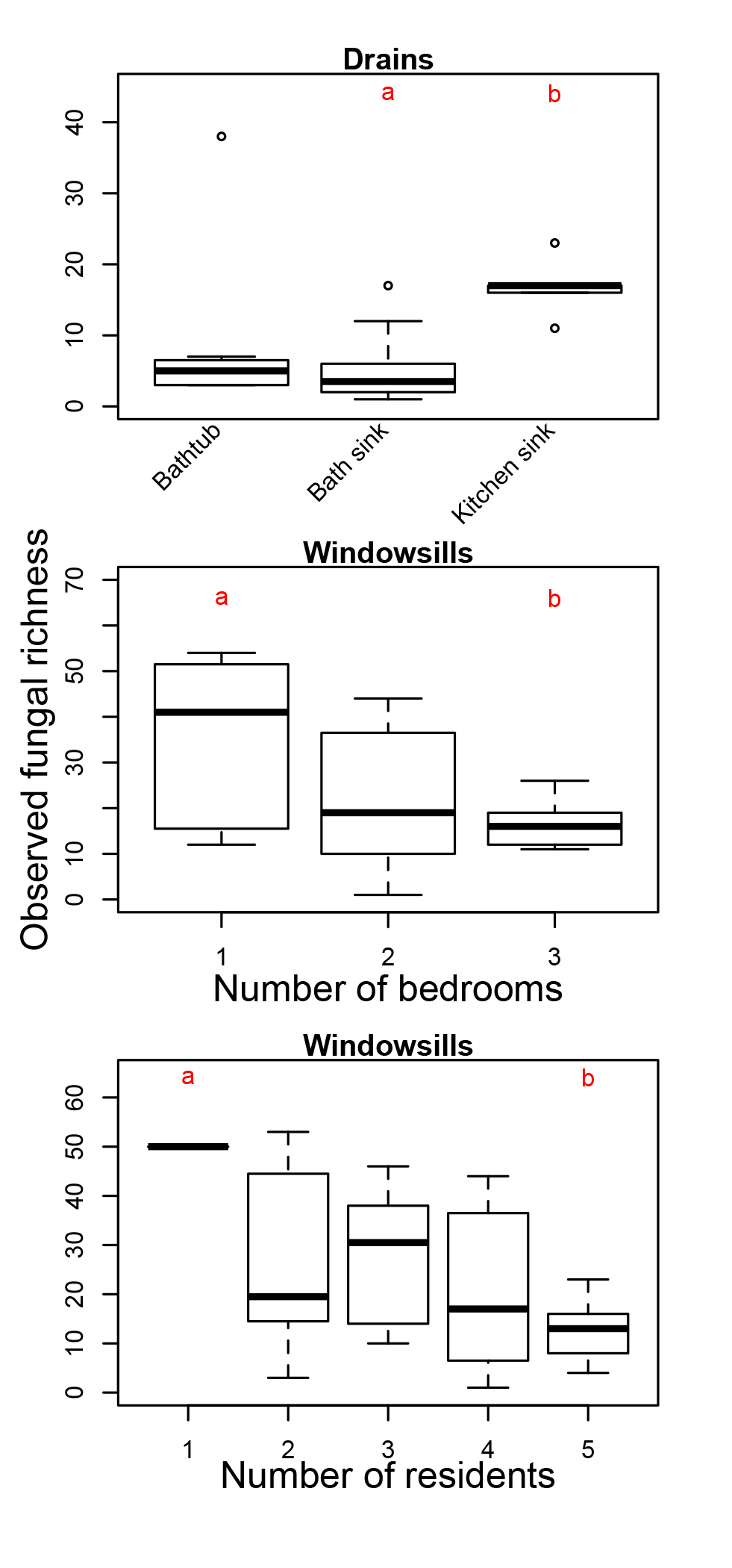

Supplement: Figure S3 — Observed differences in fungal OTU richness within surface types across different seasons, room locations, unit characteristics, and resident behavior. Shown are the significant factors affecting fungal richness on drains and windowsills. There were no differences observed for skins. The factors included: unit, room type, season, number of bedrooms, number of bathrooms, number of residents, age of unit, whether a humidifier was occasionally used, and whether a houseplant(s) were present. Within drains, bathroom sinks were less rich than kitchen sinks (anova – Table S3). Fungal richness of windowsills decreases with increasing number of bedrooms (anova, df = 2, Fvalue = 2.88, p = 0.06) and the number of residents (anova, df = 4, Fvalue = 2.77,p = 0.04). Letters indicate significant differences according to post-hoc Tukey Honest Significant Differences (p<0.05). Nonsignificant trends were based on tests of anova, p>0.05. (TIF) [file pone.0078866.s003.tif]

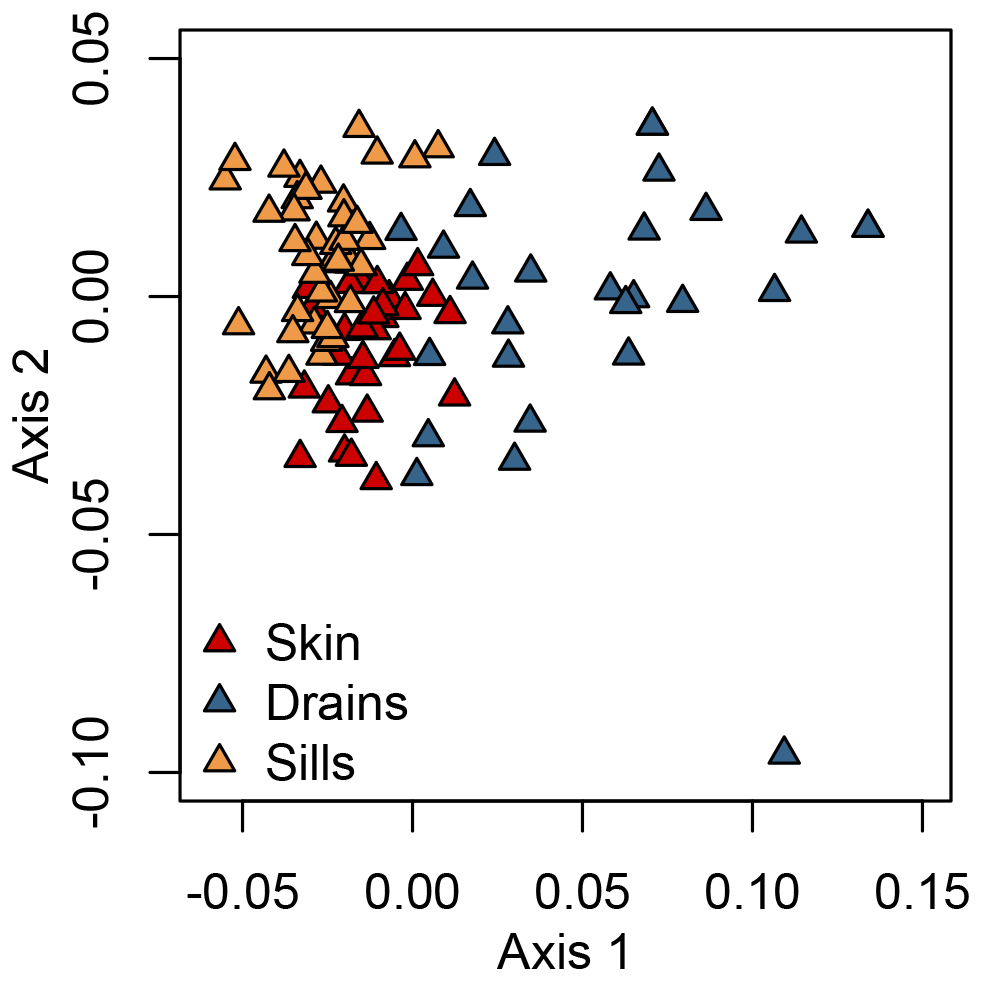

Supplement: Figure S4 — Visual representation of the fungal composition on different surface types using nonmetric multidimensional scaling (NMDS) based on the presence-absence of taxa (Jaccard index). As with the abundance-based data Morisita-Horn index, the three surface types cluster within types, with greater dispersion within drains. (TIF) [file pone.0078866.s004.tif]

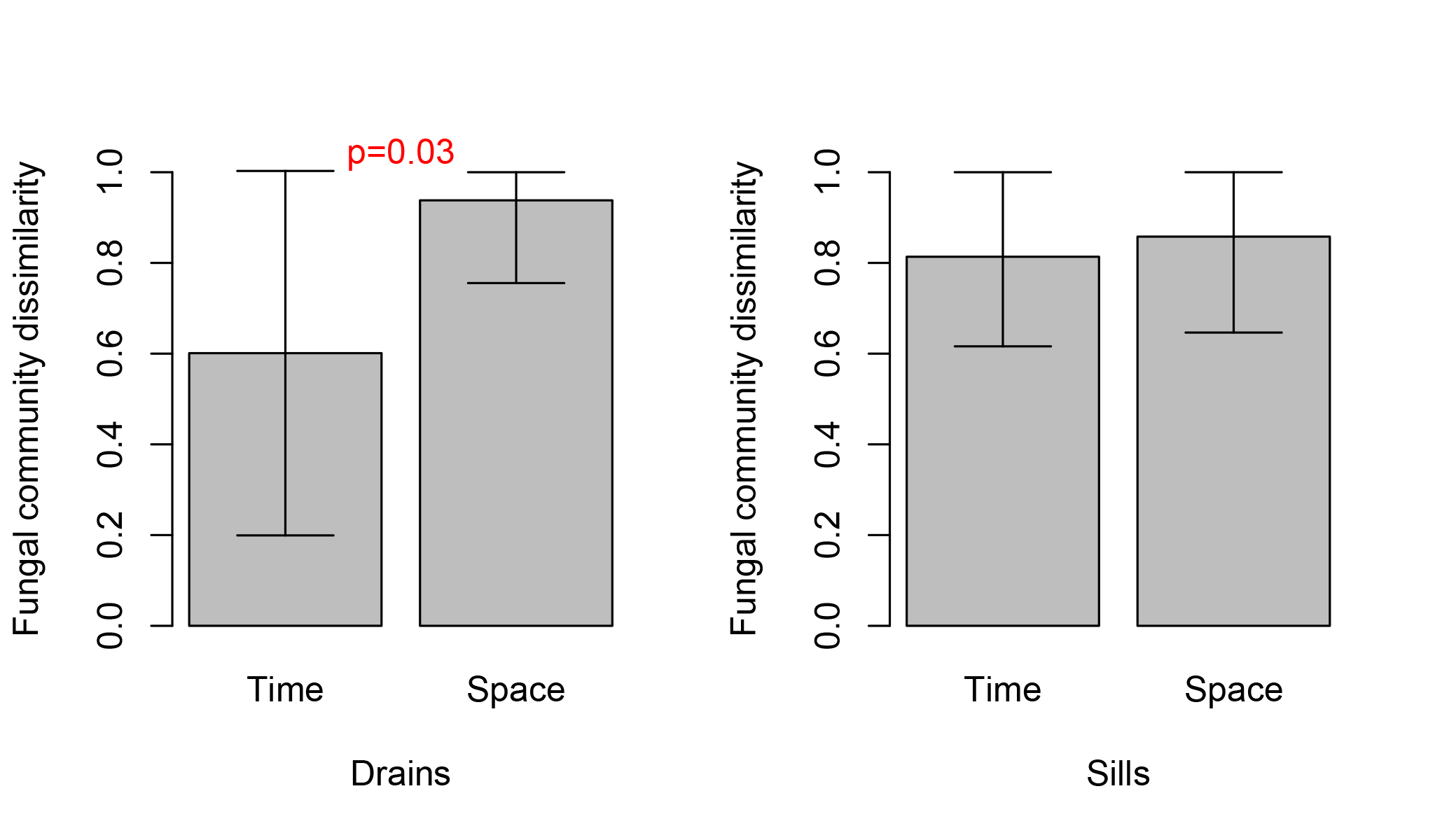

Supplement: Figure S5 — Variation of fungal community composition on drains and windowsills across time and space. For drains, “time” is the mean dissimilarity in fungal community composition within a drain across seasons, while “space” is the dissimilarity across all drains within a season. Likewise for windowsills. Fungal communities within drains show more continuity over time than fungal communities on windowsills (2-sample t-test for drains: t = −2.62, df = 8.15, p = 0.03; for windowsills: t = −0.5, df = 14.25, p = 0.62). (TIF) [file pone.0078866.s005.tif]
