# Supplementary material for: The Diversity and Distribution of Fungi on Residential Surfaces
Source: PLoS One. 2013 Nov 1;8(11):e78866. doi: 10.1371/journal.pone.0078866 (PMC3815347; doi:10.1371/journal.pone.0078866)
Supplement: Table S2 — Fungal OTUs identified to genera in negative controls and removed from the study community table prior to analysis. Taxa marked with * were present in greater than 25% of all negative controls (n = 5), and taxa marked with ? were present as 1% or greater of total negative control sequences (n = 13). (DOCX) [file pone.0078866.s007.docx]

|  |  | GenBank ID | Database Identity | Blast Match (%) |
| --- | --- | --- | --- | --- |
| 1 | OTU_56^ | JN032539 | uncultured_fungus | 98.67 |
| 2 | OTU_62^ | FJ475553 | uncultured_Myxotrichaceae | 98.67 |
| 3 | OTU_51^ | AB232884 | Coccidioides_posadasii | 100 |
| 4 | OTU_139^ | HM069362 | uncultured_fungus | 88.11 |
| 5 | OTU_223^ | EU918705 | Sordaria_humana | 100 |
| 6 | OTU_152^ | HM488536 | uncultured_Helotiales | 98.01 |
| 7 | OTU_219^ | FN565275 | uncultured_Helotiales | 98.64 |
| 8 | OTU_249^ | AB232883 | Coccidioides_posadasii | 84 |
| 9 | OTU_4*^ | KC464335 | Aureobasidium_pullulans | 100 |
| 10 | OTU_114*^ | JQ781717 | Penicillium_brevicompactum | 100 |
| 11 | OTU_68*^ | HQ914284 | Rhizopogon_salebrosus | 99.33 |
| 12 | OTU_304^ | AB232883 | Coccidioides_posadasii | 88.67 |
| 13 | OTU_310^ | AM902038 | uncultured_Ascomycota | 97.33 |
| 14 | OTU_5 | JQ946390 | Cladosporium_cladosporioides | 100 |
| 15 | OTU_95 | KC254055 | Hanseniaspora_uvarum | 100 |
| 16 | OTU_316 | FN610859 | uncultured_fungus | 99.33 |
| 17 | OTU_560 | JX270369 | Mortierella_sp_04NY01 | 94.23 |
| 18 | OTU_9 | FJ757371 | uncultured_fungus | 96.67 |
| 19 | OTU_365 | JX270557 | Penicillium_sp_20KY03 | 100 |
| 20 | OTU_279 | JF497125 | uncultured_fungus | 96.05 |
| 21 | OTU_388 | AB175249 | Thysanophora_penicillioides | 99.33 |
| 22 | OTU_6* | AB488490 | Exophiala_sp_NH1238 | 100 |
| 23 | OTU_31 | JQ966756 | Rhodotorula_mucilaginosa | 100 |
| 24 | OTU_3 | FJ757025 | uncultured_fungus | 100 |
| 25 | OTU_408 | JN098110 | fungal_sp_19S_6_2 | 100 |
| 26 | OTU_598 | FJ808062 | uncultured_yeast | 95.36 |
| 27 | OTU_963 | JN133556 | Trichoderma_ghanense | 100 |
| 28 | OTU_29* | JN634833 | Alternaria_tenuissima | 100 |
| 29 | OTU_154 | NR_073357 | Cryptococcus_cyanovorans | 98.01 |
| 30 | OTU_281 | JN905839 | uncultured_fungus | 100 |
| 31 | OTU_54 | KC152884 | Malassezia_globosa | 100 |
| 32 | OTU_39 | GQ509327 | uncultured_fungus | 100 |
| 33 | OTU_465 | JN906659 | uncultured_fungus | 95.95 |
| 34 | OTU_1014 | EU807123 | uncultured_soil_fungus | 98.67 |
| 35 | OTU_595 | EF521203 | uncultured_fungus | 100 |
| 36 | OTU_703 | JN019831 | Cryptococcus_amylolentus | 97.2 |
| 37 | OTU_677 | HQ211781 | uncultured_Venturia | 97.33 |
| 38 | OTU_891 | FJ903326 | Sarea_sp_C65 | 98.68 |
| 39 | OTU_16 | HF947078 | uncultured_Cladosporium | 100 |
| 40 | OTU_109 | JX421701 | Lewia_infectoria | 100 |
| 41 | OTU_12 | KC464342 | Epicoccum_nigrum | 100 |
| 42 | OTU_191 | KC152904 | Cryptococcus_diffluens | 100 |
| 43 | OTU_107 | KC254020 | Cryptococcus_albidus | 100 |
| 44 | OTU_98 | AB811857 | Trametes_versicolor | 100 |
| 45 | OTU_130 | JX029950 | Cortinarius_sp_H43 | 100 |
| 46 | OTU_652 | JN594608 | Golovinomyces_biocellatus | 94.59 |
| 47 | OTU_865 | FJ553396 | uncultured_Herpotrichiellaceae | 85.21 |
| 48 | OTU_759 | AM999757 | uncultured_fungus | 100 |
| 49 | OTU_994 | DQ421309 | uncultured_soil_fungus | 91.78 |
| 50 | OTU_13 | HF947073 | uncultured_Cladosporium | 100 |
| 51 | OTU_278 | JN904620 | uncultured_fungus | 99.33 |
| 52 | OTU_128 | JX624271 | Cladosporium_sp_JDH3 | 99.32 |
| 53 | OTU_718 | JX270435 | Debaryomyces_sp_10NY11 | 96.77 |
| 54 | OTU_104 | JQ666340 | uncultured_soil_fungus | 100 |
| 55 | OTU_466 | EU573019 | Phoma_plurivora | 99.32 |
| 56 | OTU_40 | KC152885 | Malassezia_restricta | 100 |
| 57 | OTU_497 | EF488438 | Peniophora_sp_XL_A26 | 99.32 |
| 58 | OTU_171 | NR_103569 | Phialocephala_fluminis | 95.59 |
| 59 | OTU_111 | KC119199 | Aspergillus_fumigatus | 100 |
| 60 | OTU_10 | JX915255 | Fusarium_oxysporum | 100 |
| 61 | OTU_314 | JX042766 | uncultured_ectomycorrhizal_fungus | 100 |
| 62 | OTU_987 | GQ999317 | uncultured_fungus | 96.03 |
| 63 | OTU_689 | JN906112 | uncultured_fungus | 100 |
| 64 | OTU_536 | EU819476 | Laccaria_amethystina | 100 |
| 65 | OTU_883 | GU366729 | uncultured_fungus | 100 |
| 66 | OTU_362 | AF090856 | Battarrea_phalloides | 98.54 |
| 67 | OTU_540 | FN610873 | uncultured_fungus | 94.74 |
