# Supplementary material for: The Diversity and Distribution of Fungi on Residential Surfaces
Source: PLoS One. 2013 Nov 1;8(11):e78866. doi: 10.1371/journal.pone.0078866 (PMC3815347; doi:10.1371/journal.pone.0078866)
Supplement: Table S3 — Simpson and Shannon diversity indices for fungal richness on the different surface types. As with observed richness, drains are less diverse than sills and skin, but all three are significantly different from each other (anova: Simpson: df = 2, F-value = 24.53,p<0.01; Shannon: df = 2,F-value = 33.29,p<0.01; post-hoc TukeyHSD pairwise comparisons p<0.01). Bathroom sinks are less diverse than kitchen sinks (anova: Simpson: df = 2, F-value = 5.05,p = 0.01; Shannon: df = 2,F-value = 6.16,p<0.01; post-hoc TukeyHSD pairwise comparison between bathroom sinks and kitchen sinks p<0.01). (DOCX) [file pone.0078866.s008.docx]

|  | Simpson | Shannon |
| --- | --- | --- |
| Skin - all | 0.88 | 2.86 |
| Sills - all | 0.70 | 2.05 |
| Drains - all | 0.43 | 1.03 |
|  |  |  |
| Bathroom sink | 0.29 | 0.62 |
| Bathtub drain | 0.54 | 1.17 |
| Kitchen sink | 0.72 | 1.90 |
