# Supplementary material for: The Diversity and Distribution of Fungi on Residential Surfaces
Source: PLoS One. 2013 Nov 1;8(11):e78866. doi: 10.1371/journal.pone.0078866 (PMC3815347; doi:10.1371/journal.pone.0078866)
Supplement: Table S4 — Predictive factors of fungal community composition on surfaces within residential apartments based on the (presence-absence) Jaccard Index, after the plate effect of the three sequencing runs were factored out. (DOCX) [file pone.0078866.s009.docx]

|  | All |  | Drains |
| --- | --- | --- | --- |

| n | 101 |  | 28 |
| --- | --- | --- | --- |

|  | df | F-value | R^2^ | p |  | df | F-value | R^2^ | p |
| --- | --- | --- | --- | --- | --- | --- | --- | --- | --- |
| Type^1^ | 2 | 3.3 | 0.06 | 0.001 |  | 2 | 1.9 | 0.11 | 0.003 |
| Residential Unit | 10 | 1.4 | 0.13 | 0.001 |  | 10 | 1.7 | 0.47 | 0.001 |
| Room function | 3 | 1.2 | 0.03 | 0.001 |  | -- | -- | -- | ns |
| Residuals | 83 |  | 0.74 |  |  | 14 |  | 0.40 |  |
|  |  |  |  |  |  |  |  |  |  |

|  | Sills | | | |  | Skin | | | |
| --- | --- | --- | --- | --- | --- | --- | --- | --- | --- |
| n | 41 | | | |  | 32 | | | |
|  | df | F-value | R^2^ | p |  | df | F-value | R^2^ | p |
| Residential Unit | 10 | 1.4 | 0.31 | 0.001 |  | 8 | 1.2 | 0.29 | 0.001 |
| Room function | 3 | 1.2 | 0.08 | 0.07 |  | -- | -- | -- | --^2^ |
| Residuals | 26 |  | 0.56 |  |  | 21 |  | 0.62 |  |
|  |  |  |  |  |  |  |  |  |  |
