# Supplementary material for: The Diversity and Distribution of Fungi on Residential Surfaces
Source: PLoS One. 2013 Nov 1;8(11):e78866. doi: 10.1371/journal.pone.0078866 (PMC3815347; doi:10.1371/journal.pone.0078866)
Supplement: Table S5 — Mantel correlations between geographic distance and fungal community distance. (DOCX) [file pone.0078866.s010.docx]

|  | n | r | p |
| --- | --- | --- | --- |
| Drains |  |  |  |
| Summer | 15 | 0.13 | 0.10 |
| Winter | 13 | -0.02 | 0.50 |
| Sills |  |  |  |
| Summer | 25 | 0.10 | 0.06 |
| Winter | 15 | 0.32 | **0.01** |
| Skin |  |  |  |
| Summer | 17 | 0.25 | 0.15 |
| Winter | 15 | 0.46 | **0.01** |

Notes: Significant values in bold.
