# Supplementary material for: The Diversity and Distribution of Fungi on Residential Surfaces
Source: PLoS One. 2013 Nov 1;8(11):e78866. doi: 10.1371/journal.pone.0078866 (PMC3815347; doi:10.1371/journal.pone.0078866)
Supplement: Protocol S1 — Processing reads with QIIME and comparisons with UPARSE. (DOCX) [file pone.0078866.s011.docx]

Protocol S1: Processing reads with QIIME and comparisons with UPARSE

The three separate runs were denoised in an effort to remove spurious sequences resulting from sequencing error [[1](#_ENREF_1)]. The three runs were then combined, and all sequences outside of a common length (200-500bp) were excluded. The remaining sequences were clustered into operational taxonomic units (OTUs) using the UCLUST method [[2](#_ENREF_2)]. We did not check for chimeras, as the targeted region (i.e. ITS1) does not contain conserved regions that are in general the dominant placement for chimeric breakpoints [[3](#_ENREF_3)]. Fungal taxa present in nine negative controls were excluded from the study samples. Fungal taxa appearing only once (i.e. singletons) were also removed from the community table. Finally, we only included those samples that contained at least 100 sequences.

The total number of OTUs was determined to be slightly higher (n=1,305) than with UPARSE (n=966). The distribution of the decrease in OTUs was not consistent across samples, such that almost as many samples gained OTUs under UPARSE relative to QIIME as did samples loose OTUs. The fungal composition and relationship among samples were unchanged under the two analyses. The implementation of UPARSE was faster than the QIIME protocol because of the absence of the lengthy denoising step implemented in QIIME.

1. Reeder J, Knight R (2010) Rapidly denoising pyrosequencing amplicon reads by exploiting rank-abundance distributions. Nat Methods 7: 668-669.

2. Edgar RC (2010) Search and clustering orders of magnitude faster than BLAST. Bioinformatics 26: 2460-2461.

3. Porazinska DL, Giblin-Davis RM, Sung W, Thomas WK (2012) The nature and frequency of chimeras in eukaryotic metagenetic samples. J Nematol 44: 18-25.
